# It is over three decades of graduate education in Epizootiology at the University of Ibadan, Nigeria (1975–2011): is there a need to revise the curriculum?

**Published:** 2012-07-12

**Authors:** Babasola Oluseyi Olugasa, Ighodalo Folorunso Ijagbone, Gabriel Oluwole Esuruoso

**Affiliations:** 1 Department of Veterinary Public Health and Preventive Medicine, University of Ibadan, Ibadan, Nigeria; 2 College of Agriculture and Sustainable Development, Cuttington University, Suakoko, Liberia; 3 Nigerian Institute of Science Laboratory Technology, Samonda, Ibadan, Nigeria; 4 VetAcademic Resource Foundation, New Bodija Estate, Ibadan, Nigeria

**Keywords:** Ecosystem Health, epizootiology, curriculum, Global Health, International Programme

## Abstract

Epizootiology is the study of variable factors, events, forces and circumstances that contribute to the occurrence, distribution, control and prevention of ill-health, diseases and other problems in animal groups. It is a key component of veterinary medicine education at the University of Ibadan, Nigeria since 1975. It started as a Graduate Certificate in Epizootiology (GCE) in 1976. Later it was revised into M.Sc. Epizootiology in 1986. At graduate level, epizootiology curriculum has supported the M.Sc. Epizootiology programme. It compliments training in Veterinary Public Health and Preventive Medicine. This epizootiology curriculum has been operational at graduate level for more than three decades. Now in 2011, a consortium of English speaking West African Universities is committed to review the current curriculum at the University of Ibadan to strengthen health systems in an interdependent world with scope for internationalized practicum in disease investigation. Emphases are made towards skills development in molecular studies on disease causal agents and the mapping of associated geographic risk factors, including indigenous knowledge and practices. It is notable that most English-speaking West African countries including Ghana, Liberia, Sierra Leone and Gambia either lack a Veterinary School or just started some, but do not have graduate programme in Epizootiology. Thus, the curriculum at Ibadan is positioned to make impact in three key areas, namely, sub-regional ecosystem health studies, improving human-animal disease surveillance programmes, and in indigenization of bio-technology for monitoring and evaluation of trans-boundary animal disease control interventions for global health in West Africa.

## To the editors of the Pan African Medical Journal

The graduate training in Epizootiology started at the University of Ibadan in 1976 as Post-Graduate Diploma [1–4]. After ten years, it emerged into Master of Science in Epizootiology. Contemporary programmes in veterinary epidemiology have made emphasis towards veterinary economics as an area of focus in training, thus called veterinary epidemiology and economics [1, 2]. Epizootiology programme at Ibadan however makes emphases to both social and economic jurisprudence, thus supporting veterinary public health and preventive medicine training respectively. In 2001, participatory epizootiology as well as ecologic and geographic information systems technology were introduced [4] into the studies and areas of emphases of epizootiology at the University of Ibadan. The two new areas of emphasis in epizootiology at Ibadan were stimulated by the emergence of African swine fever in Ibadan and environs in the south-western Nigeria in 2001 as a major trans-boundary animal disease (TAD) [5–7]. Coupled with the lingering need for containment of endemic, emerging and neglected zoonoses in West Africa [8], especially in the events of swine and avian influenza epidemics [9, 10] were rabies [11–13], Lassa and Yellow fever as well as bovine tuberculosis which were human-animal diseases (zoonoses) that had further competed for the attention of teachers, administrators, practitioners, public opinion leaders and graduate students in the overriding applications given to graduate training programme in epizootiology. Thus, while the coursework in epizootiology had aided the recognition of the increasing role and importance of Veterinary Public Health and Preventive Medicine within Nigeria [1, 2] and the integrated development of human-animal diseases and ecosystem health programmes at the University of Ibadan, it could also be observed that some potentials of the discipline of epizootiology had not been significantly utilized to enable development of manpower in this unique inter-disciplinary field that is capable of international applications beyond the infectious diseases in community and regional planning for ecosystem health promotion in West Africa [6, 12, 14, 15]. Thus, the West African sub-region has much to achieve in harnessing emerging potentials.

Few West African countries emerging from prolonged civil war had also experienced interrupted higher education, especially in human and animal health, with high risk of endemic, emerging and neglected zoonoses, along with high economic impact trans-boundary animal diseases (TADs). Where there are no significant numbers of veterinarians, a graduate programme of M.Sc. Epizootiology is uniquely placed to mentor the emerging generation of post-war college graduate students in the sub-region to play active and vital role in the vanguard of sustainable development, ecosystem health, animal and crop agriculture, food security, wildlife conservation, increasing local production and use of proteins from animal sources.

Each of the three authors of this paper has played unique roles in the development, contemporary application and evolution of graduate programmes in epizootiology at the University of Ibadan. This paper gives some fundamental explanations on the need for the revision of the existing curriculum. Professor Gabriel Oluwole Esuruoso who designed and presented the Graduate Certificate and M.Sc. Epizootiology programmes for approval by the Senate of the University of Ibadan in 1975 also taught most of the required courses at inception [1–4], and supervised more than 40 dissertations and theses in the Department of Veterinary Public Health and Preventive Medicine. The co-authors on this paper are two of his former students on M.Sc. Epizootiology degree programme who are now career status lecturers in the same department at the University of Ibadan. The authors agree in the affirmative that there is a need to revise the curriculum to address the emerging and perceived needs. We also indicate the direction that is critical for the revision, providing some fundamental thematic areas of emphases, and a role for inter-university collaboration in West Africa to foster a critical mass of scholars in the various areas, utilizing opportunities for credit transfer and joint supervision of projects where practicable. These were considered by the authors in charting a way forward to strengthen internationalization of epizootiology programme at the University of Ibadan within a consortium of Universities addressing the obvious gap in available manpower for TADs and zoonoses studies and containment promotion in West Africa. A revised M.Sc. Epizootiology curriculum for improving graduate programmes in the surveillance of human-animal diseases is critical for global health capacity building among developing countries of West Africa.

### A unique platform for Nigeria field epidemiology competence

The Department of Veterinary Public Health and Preventive Medicine at the University of Ibadan was establishment in 1975 and developed a Post-Graduate Certificate (PGC) course in Epizootiology. Thus, the PGC in Epizootiology enrolled 3 students in 1976. Training support was received from the young faculty at the Veterinary School and inputs from faculties in the Medical School and the University College Hospital, Ibadan [1]. Thus, the appreciation of “one-health” initiative [16] was very early at the University of Ibadan. Being aware of this premise, the Centers for Disease Control and Prevention (CDC) Consultant on the N-FELTP programme (himself a graduate of Doctor of Veterinary Medicine programme at the University of Ibadan) was smart to harness this platform to bring on board for the first time the Veterinary Epidemiology Track into the Master of Public Health degree programme on the N-FELTP [17]. This therefore made historic landmark as the first programme with the three tracks of physician (field), veterinarian (veterinary epidemiology) and public health laboratorian (laboratory management) tracks.

The PGC in Epizootiology was upgraded into M.Sc. in Epizootiology ten years later in 1986. Albeit, a multi-disciplinary course with a central 3 unit course on Advanced Epizootiology, while students selected from a list of recommended elective and compulsory courses to adapt available modules for targeted skills and competencies. At that period, a four-unit course on Advanced Epizootiology served the two ends of a Master of Veterinary Public Health (MVPH) and Master of Preventive Veterinary Medicine (MPVM) degree programmes that were offered in the same department. Essentially, the department had the latter two as the end in view, based on situations in Nigeria and the perceived veterinary manpower needs at that time. By 2006, there had been seven graduates of the M.Sc. Epizootiology degree programme, each with unique blend of academic background, skills and career emphasis. This had been the only programme in Epizootiology or Veterinary Epidemiology in Nigeria until the recent emergence in 2011 of a new curriculum for M.Sc. Veterinary Epidemiology at Ahmadu Bello University, Zaria, Nigeria with support from the MacArthur Foundation. The first two of the authors of this review article are among the seven graduates of the M.Sc. Epizootiology programme at the University of Ibadan, Nigeria.

Efforts to foster a bioscience cluster in Ibadan for high-skills in ecosystems health have received the special academic link support from the University of Ibadan to Drs. Ighodalo Ijagbone and Babasola Olugasa for scholar's exchange to build a Canadian-Nigerian biotechnology research bridge in animal health and epizootiology. A series of lectures in Epizootiology “ that covered investigating and managing trans-boundary animal diseases” with a specific case on “dying pigs due to African swine fever”, and “demystifying diagnostic kits production within graduate residency in epizootiology at the University of Ibadan” were popularly attended and passionately supported by graduate students and the faculty. Subsequently, an initiative in higher education for strategic revision of the M.Sc. Epizootiology curriculum was put up at the University of Ibadan in 2010 by a team of five scholars (who are teachers of veterinary medicine courses at Ibadan) under the leadership of the first author of this paper to the John D. and Catherine T. MacArthur Foundation. Evidently, there is a strong consensus building underway to emerge a robust trans-disciplinary curriculum in epizootiology at Ibadan. The coast is clear and the tide is strong.

### Why build high-skills in ecosystem health?

The University of Ibadan along with some of the oldest Universities in West Africa is set to embark on a review of its epizootiology curriculum to include training in competencies for ecosystem health in West Africa. This will place the ecology of West Africa in the balance [16]. The lead university is the University of Ibadan (oldest University in Nigeria that was established in 1948) and in collaboration with the University of Sierra Leone (oldest University in West Africa, formerly Fourah Bay College, established in 1827), Cuttington University, Liberia (1889-third oldest University in West Africa), University of Ghana (established in 1948 and oldest University in Ghana), and Njala University, Sierra Leone (1964). Together with the National Veterinary Research Institute, Vom, Nigeria, Nigerian Institute of Science Laboratory Technology, the College of Medicine, University of Ibadan, the National Zoonoses Center, Nigeria, the Ghana Veterinary Medical Association, and the Nigerian Veterinary Medical Association. This consortium seeks to jointly offer cocktail input into the revision of the curriculum for professionalism in ecosystem health as key competence in M.Sc. Epizootiology. The West African sub-region with a number of post-war countries and resource limited capacity to combat endemic, emerging and neglected zoonoses is cardinal in global health initiative for safer world and cities in the present century. The sub-region is significant in number of neglected communities with neglected diseases, including rabies [11–13], and emerging influenza A viruses [9, 10], underscoring the adage of the strength of a chain being its weakest link.

Epizootiology curriculum needs to address the issue of climate change in relation to disease distribution. The need to train students to conduct research across geographical boundaries in West Africa within a trans-disciplinary milieu is also to address competence in spatial analysis. For example, the rapid encroachment of the desert has led to an unprecedented expansion of what we call derived Savannah in Nigeria in the past few years. This has significantly affected the vegetation of Nigeria and necessarily led to the emergence of new pathogens and changes in agricultural practices in the country in the past 3 decades. Indeed, the derived Savannah area in Nigeria is one of the most practical demonstrations of how climate change modulates disease epidemiology as a result of changes in the ecosystem that supports vectors and thus affects the abundance and distribution of vectors. The scarcity of in-country training and research activities addressing these issues in the past decades has led to loss of considerable amount of data on the practical relationship between ecosystem change resulting from climate change and the spatial distribution of tropical diseases [8–12].

Beyond animal and human health issues that are receiving greater attention in Ibadan at the moment with relatively strong teaching and research foci in preventive veterinary medicine and veterinary public health, is ecosystem health studies for environmental planning and sustainable rural development. This includes energy balance and wildlife health. Hitherto, these aspects have not received adequate attention in the existing curriculum. The M.Sc. Epizootiology is strategically positioned to support trans-disciplinary initiative to which general biologists and social scientists could be mentored towards competence in ecosystem/environmental health planning. Adequate revision will package basic aspects of ecosystem quality surveillance into the proposed curriculum to strengthen training in West African Universities.

### Building skills in spatial analysis, environmental health and sub-regional planning

For the first time, we are introducing a program that orientates trainees to spatial epizootiology using Geographic Information System (GIS), an area that is often neglected in graduate training in most parts of the world [6, 12, 14]. GIS is an exciting technology which is becoming a more common part of everyday life, and which biologists explore for spatial evaluation of disease ecology, the host-parasite-environment components, ecosystem dynamics, pathogen distribution pattern and risk management planning. The use of Global Positioning Systems (GPS) to collect geo-referenced data about ecosystem attributes also captures data about the climate and its association with disease patterns [6, 12, 14]. The training will follow an integrated approach linking human, animal and ecosystem health traversing the derived Savannah, rain forest, mangrove and swampy areas of West Africa in order to improve TADs control strategies, ecosystem sustainability, food security and rural development. The M.Sc. Epizootiology curriculum normally consists of compulsory (core) and elective modules, in addition to a dissertation. It is hoped that part of the core module will now be directed at spatial epizootiology and ecosystem information analysis for international projects, public health administration and policy making reforms.

We believe that graduates of the proposed programme will be located in several parts of Nigeria, Ghana, Liberia, Sierra Leone and the Gambia and, having been sensitized to the ecosystem approach to disease surveillance, will form a strong network for future research and collaborative efforts that focus on the impact of climate change on trans-boundary animal diseases and ecosystem health in West Africa.

### Reinventing a robust curriculum

The focus of graduate curriculum review in epizootiology at the University of Ibadan is to add value to academic training, including research and mentoring opportunities for young women and men on career track in the sub-region. This will be achieved through the provision of hands-on practicum on detection, prevention and containment of TADs, using selected hotspots of endemic and neglected diseases in Nigeria, Ghana, Liberia, Sierra Leone and the Gambia. The proposed graduate programme focuses on training of manpower that will effectively understand the state-of-the-arts in TADs detection, prevention, and containment systems, and be able to utilize the system efficiently in West Africa.

Deficiency about current training and research includes reduced research synergy and teamwork that is focus on strategic operations for TADs prevention and containment. There is need to foster active and strategic research agenda to drive mentoring and services delivery efforts and direction in this milieu. It is this deficiency that a revision of the curriculum is needed to address. One major setback to surveillance of TADs in West Africa is the overbearing cost of diagnostic reagents. This is a thematic area of emphasis in the research and development component of the curriculum.

The proposed program will therefore provide practical disease prevention tools for trainees. Increasing biological risk management on the farm is the most important thing an animal producer can do to protect animals from high economic consequence TADs and to protect the public from zoonotic diseases. The faculty will mentor graduate students to develop a comprehensive set of practical disease management tools for veterinarians, public health practitioners and animal producers to be built around the routes of disease transmission. One set of tools each will be specifically designed for service providers to use with swine, poultry, dog and cat, cattle, sheep and goats clients. Animal Health Officers will be able to access these from an online database of biological risk management assessment questions and create a question set based on the type of farm being evaluated. Then they would conduct a walk through assessment on the farm and enter the results on a checkboard. A report of the most vulnerable routes of disease transmission will be generated along with recommendations to enhance infection risk management. The Animal Health Official will then work with the producer or community to prioritize and implement the recommendations. These tools will be available in English and three major West African local languages. Materials include information handouts, disease fact sheets, farm signs, economics of risk management, and how to protect production units if a zoonotic disease is diagnosed.

Under-utilization of the trans-disciplinary nature of the M.Sc. Epizootiology program has lead to marginal collaborative teaching and research within Nigeria and across Ghana, Liberia and Sierra Leone that is committed to building the regional capacity for TADs containment. Among the limited number of graduates of the current M.Sc. Epizootiology programme is a technologist that proceeded to develop diagnostic kit through indigenization of biotechnology in Nigeria and is currently the Registrar and Director-General of the Nigerian Institute of Science Laboratory Technology. Another graduate of the current programme combined courses in Industrial and Production Engineering with the biomedical courses in veterinary medicine and zoonoses and was actively engaged in Technical Cooperation among Developing Countries in the assistance for prevention and containment of TADs in West Africa.

**Figure 1:**
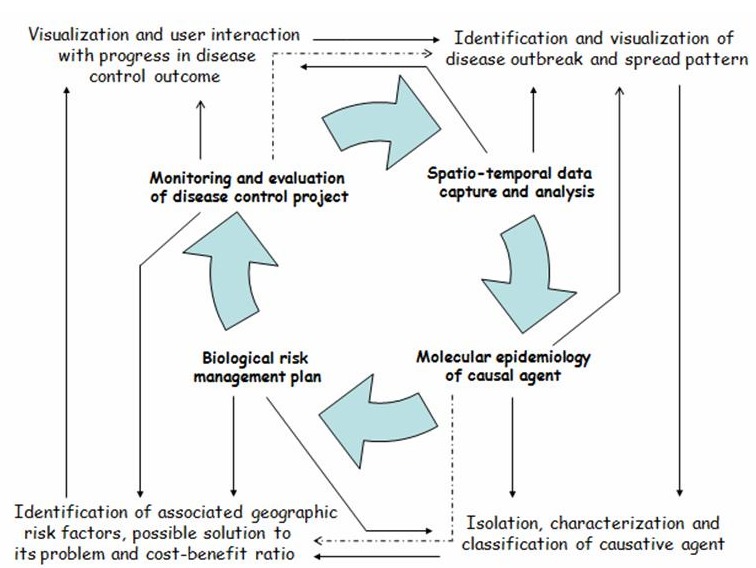
Proposed areas of emphasis in revised graduate curriculum for M.Sc. Epizootiology at the University of Ibadan, Ibadan, Nigeria Current curriculum for eqizootiology places some emphases on isolation, characterization and classification of causal agents; identification of associated geographic risk factors, possible solution options to problems and cost-benefit ratio of control options. A strategic revision will add training for competence in analytical planning and visualization with Geographic Information Systems (GIS), such that an individual with M.Sc. Epizootiology degree is capable of investigating a problem in spatio-temporal pattern, and design biological risk management plan for it. Graduates will be able to present in visual map progress made in disease control projects at national and international levels in West Africa. Development of skills in conducting molecular evalution of the causal agent is additional competence

The proposed curriculum will in the end fit into the One Health concept. The One Health concept is a world-wide strategy for expanding interdisciplinary collaborations and communications in all aspects of health care for humans, animals and the environment. The synergism achieved will advance health care for the 21st century and beyond by accelerating biomedical research discoveries, enhancing public health efficacy, expeditiously expanding the scientific knowledge base, and improving medical education and clinical care [16]. When properly implemented, it will help protect and safe untold millions of lives in our present and future generations. The core areas of emphases of the programme are (i) spatio-temporal data capture and information analysis, (ii) diagnostic techniques and molecular epizootiology, (iii) biological risk management planning, and (iv) use of indicator in the monitoring and evaluation of disease control projects [7] (Figure 1).

### Current interest in the M.Sc. Epizootiology programme

The M.Sc. Epizootiology course suffered limited advertisement, being mainly within the veterinary domain. Admissions have been so small (Table 1). Its potentials have not been well popularized. Coupled with this, are the obvious under funding challenges of higher education in Nigeria, which have resulted in limited field and laboratory training, making the course unattractive. It is therefore anticipated that with the robust publicity machinery that will be put in place, a review of the course, its enrichment with quality field and bench practicum for trainees, as well as the turnout of quality graduates of the programme in the first 3 years of its performance, we envisage an increased expression of interest by potential applicants in the program. With this in mind, we expect that the new M.Sc. Epizootiology programme will be a prestigious and highly subscribed course which will attract students (both genders) willing to pay a commensurate fee to acquire the training offered (Table 2). Since this will be of benefit to both the public and private sectors, we anticipate a situation where-in these groups will oversubscribe for the limited space on the programme.

**Table 1:** Proportion of graduates in M.Sc. Epizootiology related programmes at the Department of Veterinary Public Health and Preventive Medicine, University of Ibadan, Nigeria, 2006–2010

Course	Year of Graduation	Number Graduated	Percentage (%)
M.Sc. Epizootiology	2006	0	1.9
	2007	0	
	2008	0	
	2009	0	
	2010	1	
Masters in Veterinary Public Health (MVPH)	2006	1	68.5
	2007	10	
	2008	5	
	2009	8	
	2010	13	
Masters in Preventive Veterinary Medicine (MPVM)	2006	0	11.1
	2007	1	
	2008	0	
	2009	3	
	2010	2	
Master of Philosophy (M.Phil.)	2006	0	1.9
	2007	0	
	2008	0	
	2009	1	
	2010	0	
Doctor of Philosophy (PhD)	2006	4	16.6
	2007	2	
	2008	1	
	2009	0	
	2010	2	
Total	2006–2010	54	100

M.Sc. Epizootiology was not as popularly subscribed as related programmes. This was attributed to limited emphasis projected on its potentials and subtle description as subsidiary to MVPH and MPVM.

**Table 2:** Proportion of female students enrolled in the Department of Veterinary Public Health and Preventive Medicine, University of Ibadan, Nigeria in the 2010–2011 academic session

Course	Number Enrolled	Number of Female	Percentage of Female (%)
M.Sc. Epizootiology	3	0	0
Masters in Veterinary Public Health (MVPH)	17	8	47.1
Masters in Preventive Veterinary Medicine (MPVM)	0	0	0
Master of Philosophy (M.Phil.)	1	0	0
Doctor of Philosophy (PhD)	10	4	40
**Total**	**31**	**12**	**38.7**

Ratio of female to male students enrolled in the Department of Veterinary Public Health and Preventive Medicine, University of Ibadan has been narrow over the years. Both genders are quite informed and interested in the career opportunities obtainable from some of the programmes

### Presenting opportunities, limitations and way forward

Indigenization of knowledge and of biotechnology towards surveillance and rapid diagnosis of high economic consequence TADs and zoonotic diseases are cardinal to curriculum review initiative of West African consortium of Universities. International training programmes and scholarships have allowed a wide exposure of young African scientists to Biotechnology in the last decade, meaning that the needed skills are already present on the continent. It is of note that all the principal investigators in this review project, through the John D. and Catherine T. MacArthur Foundation funding, have learned several molecular diagnostics techniques in laboratories in Europe and North America in the past few years. However, considerable bottlenecks have prevented the routinization of such techniques in laboratories in Nigeria. One of such factors is the constant need to import diagnostic kits and reagents at costs that cannot be sustained at the current level of funding available in the country. It is the aim of this revision project to create a model through which molecular biology techniques will become a routine component of disease diagnosis and surveillance at the proposed Centre for Control and Prevention of Zoonoses (CCPZ) in Ibadan. The eventual goal is to train graduates in our programme who will be able to return to their countries of origin fully equipped with the methods and processes for the indigenization of molecular and spatio-temporal techniques in the detection of zoonotic and high economic impact diseases. These individuals will be competent in the application of these techniques to community and regional planning, rural development, ecosystem health and sustainable food production in the sub-region of West Africa.

The proposed international programme in epizootiology offers opportunities for professionalism, creating an all round manpower for ecosystem health. A similar principle as operations research, work study and worksystem research in the field of industrial and production engineering is here captioned in the initiative about the proposed programme in Epizootiology. It is strategic for Ibadan to remain a custodian of global initiatives and best practices, serving the West African sub-region and sustaining its integrity. Critical limiting factors are the role of the media, migration and the moral economy of long-distance communication that is required for distance learning, credit transfer, co-supervision of split-site projects and practicum in epizootiology. The authors hope that there will be continued support from the consortium of Universities in West Africa to actualize this curriculum revision project at the University of Ibadan. The indigenization of the N-FELTP programme in Nigeria [17] and its grassroots service delivery in the continent should contribute to fostering an advancement of programmes such as this at the University of Ibadan. Therein lies the future of M.Sc. Epizootiology and ecosystem health for global health security.
